# Increased Plasma Cells and Decreased B-cells in Tumor Infiltrating Lymphocytes are Associated with Worse Survival in Lung Adenocarcinomas

**Published:** 2020-01-20

**Authors:** Hee Eun Lee, Lei Luo, Trynda Kroneman, Marie R Passow, Kristina M del Rosario, Michael R Christensen, Mary E Francis, John W O’Shaughnessy, Anthony J Blahnik, Ping Yang, Eunhee S Yi

**Affiliations:** 1 Division of Anatomic Pathology, Mayo Clinic, Rochester, MN 55905, USA; 2 Good Clinical Practice Center, Guizhou Province People's Hospital, Guiyang, China; 3 Epidemiology-Cancer Research, Mayo Clinic, Rochester, MN 55905, USA; 4 Pathology Research Core, Mayo Clinic, Rochester, MN 55905, USA

**Keywords:** Tumor-infiltrating immune cells, Tumor-infiltrating lymphocytes, Plasma cells, Adenocarcinoma, Survival

## Abstract

**Introduction:**

Clinical significance of tumor-infiltrating plasma cells and B-cells in lung adenocarcinoma is not well known.

**Methods:**

CD3, CD20 and MUM1 immunostains were performed on representative tumor blocks selected from 120 consecutive lung adenocarcinoma cases treated by surgical resection at Mayo Clinic Rochester. CD3^+^ T-cells, CD20^+^ B-cells, and MUM1^+^ plasma cells were enumerated separately in the intraepithelial (IE) compartment and the stroma (ST) by digital image analyses using whole sections. Measured tumor-infiltrating plasma cells and B-cells were correlated with patient’s overall survival (OS) using Cox proportional hazards analysis.

**Results:**

Median age of patients was 69 years (range, 46-91 years) and 52 were male. Median numbers (interquartile range) of CD20^+^ B-cells per 1mm^2^ of tumor area (IE plus ST) and IE compartment within tumor area were 590 (224-1276) and 101 (38-109), respectively; the corresponding numbers of MUM1^+^ plasma cells were 298 (180-605), and 67 (22-145), respectively. The proportion of MUM1^+^ plasma cell among all TILs (MUM1^+^ cells/[CD3^+^ cells +CD20^+^ cells+MUM1^+^ cells] × 100) ranged 1%-59% (median13%) in the tumor area and showed a significant association with OS by univariate Cox analysis (negative correlation with hazard ratio (HR)=12.50 [95% confidence interval (CI), 1.75-89.27]). There was a significant association between IE CD20^+^ B-cells and the patient’s OS in univariate analysis (positive correlation with HR=0.81 [95% CI, 0.68-0.96]). Both parameters remained significant by multivariate analysis.

**Conclusion:**

High plasma cell % among TILs in the tumor area and low IE B-cell count were associated with worse prognosis in lung adenocarcinoma patients.

## INTRODUCTION

Tumor-infiltrating lymphocytes (TILs) play an important role in anticancer immunosurveillance in a variety of human solid tumors including lung cancers [1]. Previous studies have shown that presence of various types of immune cells as well as their tissue localization may affect the clinical course in non-small cell lung cancer (NSCLC) patients [2,3]. The immune landscape of NSCLC has been extensively studied mainly on T-cell immunity that is subject to interactions of costimulatory and inhibitory cell surface proteins [4]. The inhibitory immune checkpoints are crucial to self-tolerance and immune modulation in normal physiology, but are exploited by cancer cells to promote immune resistance in the tumor microenvironment [4]. The prevalence, biologic implication and significance of humoral immunity exerted by B-cells and plasma cells in TILs are poorly understood in NSCLCs and a few studies in the literature have shown conflicting results [5-7].

In the present study, we performed a digital image analysis for B-cells and plasma cells in different compartments within the tumor using the whole sections from 120 consecutive lung adenocarcinomas that were resected at a single institution in one year (2009), and correlated with Overall Survival (OS) along with other clinical parameters for multivariate analysis as well as univariate analysis.

## MATERIALS AND METHODS

### Patients

A total of 120 primary lung cancer patients, who were treated surgically between January 1 to December 30, 2009 at Mayo Clinic (Minnesota, USA), was included in this retrospective study. All included patients had adenocarcinoma confirmed by postoperative pathology review and were drawn from a prospective hospital-based lung cancer cohort [8,9]. Detailed procedures of patient enrollment, diagnosis and treatment data collection and routine follow-up have been reported in previous studies [10,11]. All patients in present study were authorized for research and the study protocol was approved by Mayo Foundation’s Institutional Review Board.

### Immunohistochemistry

Immunohistochemistry (IHC) was performed using antibodies against CD3 (Novocastra/Leica, clone LN10, 1/250), CD20 (Dako, clone T26, 1/300) and MUM1 (Dako, clone MUM1p, 1/100) on 4 micron thick FFPE tissue sections. All antibodies were stained using the Ventana BenchMark XT platform using the following protocols; CC1 pretreatment for 32 minutes followed by antibody incubation for 32 minutes at 37°C using Ventana Optiview DAB detection (CD3) and CC1 MILD pretreatment followed by antibody incubation for 32 minutes at 37°C using Ventana Ultraview DAB detection (CD20 and MUM1).

### Digital image analysis

Slides were scanned, by a research technologist who specializes in digital imaging, at 40x magnification on the Aperio ScanScope AT Turbo brightfield instrument (Leica Biosystems) at a resolution of 0.25 microns per pixel. The images were 24-bit contiguous standard pyramid tiled TIFFs compressed via JPEG2000 with a quality setting of 70. For digital image analysis, the Aperio ImageScope Software (Leica Biosystems) was utilized and a modified nuclear algorithm was used for analysis. Image analysis was performed by a cytotechnologist.

**Figure 1:**
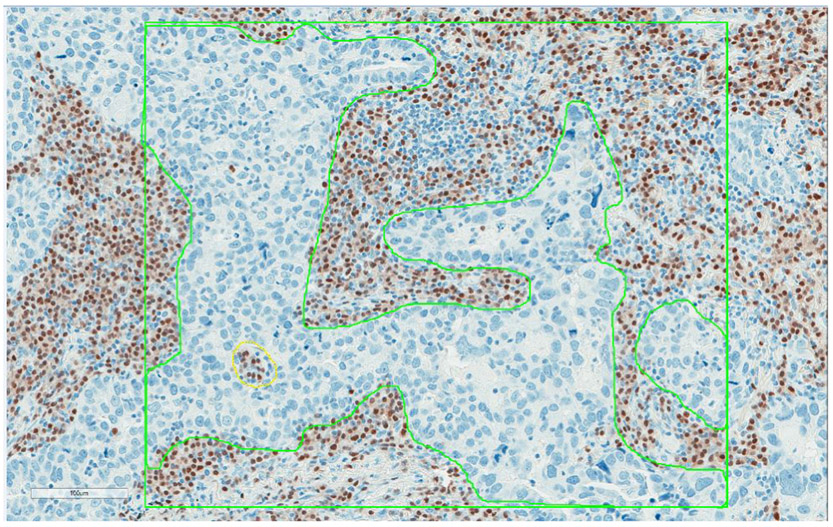
Digital image analysis of MUM1^+^ plasma cells in resected lung adenocarcinomas. The green box indicates the area measured by digital image analysis. A total of five boxes were placed on the representative areas (3 along the periphery of the tumor and 2 in the tumor center) on each case. The boxes were placed on the CD3 image first and this was used as a guide to place tiles in the same location on the CD20 and MUM1 images. A second annotation layer was added and only epithelial tumor cells were traced. Analyses were run on both (boxes and tracings) layers of annotations to get tumor-infiltrating immune cells in intraepithelial and stromal compartments of tumor separately.

Images were annotated using fixed box sizes of 600 × 500 μm^2^. A total of five boxes were placed on each image encompassing both central and peripheral portions of the tumor, in order to cover different areas of the tumor. The boxes were randomly placed on the CD3 image first and this was used as a guide to place tiles in the same location on the CD20 and MUM-1 images; CD3 was selected to minimize a staining bias. A second annotation layer was added and the tumor present in each box was traced. Analyses were run on both (boxes and tracings) layers of annotations to separate intraepithelial (IE) compartment from the entire tumor within the box that includes IE and stroma (ST) compartments (Figure 1). Data was exported into an Excel file. Counts of positive cells (membranous for CD3 and CD20; nuclear for MUM1) in the tumor area (each box; IE plus ST) or IE compartment were used for data analysis.

QC review was done on any cases that were challenging from a morphology standpoint and a random selection of cases to include a minimum of 10% of the cases by a pulmonary pathologist (ESY). The QC review included a review of the box placement and the data output (match visual assessment).

### Data description and statistical analyses

For continuous variables with non-normal distribution, the potential outliers were identified. Specifically, an outlier was considered as greater or lesser than median ± 3 × (interquartile range). Natural logarithm (ln) transformation was used to minimize the outlier. We summarized continuous variables by mean ± standard deviation and median (interquartile range), and categorical variables by frequency (%) for patient characteristics. The results were presented with original data values in survival analyses. The overall survival (OS) was defined as the time from the date of diagnosis to death from any cause or the last reported date the patient was known to be alive, until December 1, 2017. Patient who were alive, or lost to follow-up, was defined as censored in survival analyses.

Potential cut-off points to dichotomize the continuous variables with regard to OS were determined using two outcome-orientated approaches, i.e., plots of the martingale residuals and Contal and O'Quigley based on the log rank test statistic and provides corrected p values [12]. The original continuous variables were transformed into dichotomizing variables according to the potential cut-off points obtained; original continuous variables were modeled if lacking any significant cutoff point.

Univariate Cox regression was performed for evaluating the association of the prognostic factors with overall survival. Multiple Cox proportional hazard models were performed using the significant variables in univariate analysis. Hazard ratio (HR) and 95% confidence interval (CI) were calculated. Survival curves were generated by using the Kaplan - Meier method. Adjusted survival curves were created by the optimal adjustment for the covariates that were statistically significant in the Cox proportional hazard models. The p value of less than 0.1 was considered as the dichotomizing cut-off point determination and significant variables selection in the univariate Cox ’ s regression [12]. All statistical analyses were two-sided. The p value of less than 0.05 was considered significant for all statistical analyses unless otherwise specified. All analyses were performed using SAS, v.9.4 (SAS Institute Inc.). For an SAS macro of cut-off point determination was provided by Mandrekar et al. [12].

## RESULTS

### Clinical and pathologic characteristics

The age at diagnosis ranged from 46 to 91 years of age (mean and standard deviation 69.2±10.1; median 68.5). Fifty-two patients were men. Eighty-two (68.3%) patients presented with TNM stage I disease, 17 (14.2%) with stage II, 17 (14.2%) with stage III and 4 (3.3%) with stage IV. Since there were only 4 patients diagnosed with stage IV, the stage III and IV were combined in the analyses. Ninety patients (75%) were treated with surgery only and 30 patients (25%) were treated with chemotherapy and/or radiation therapy.

### Tumor-infiltrating lymphocytes

Descriptive statistics of various TILs in lung adenocarcinomas were presented along with clinical information in Table 1.

The number of each type of TILs per 1mm^2^ was represented as CD20^+^[IE], CD20^+^[IE+ST], MUM1^+^[IE], and MUM1^+^[IE+ST] depending on the compartment measured (IE vs. tumor area of IE+ST). The mean and median numbers with ranges were shown. The ratios of CD20^+^/CD3^+^ and MUM1^+^/(CD20^+^ +CD3^+^+MUM1^+^) were also calculated in [IE] and [IE+ST], respectively. MUM1^+^/(CD20^+^+CD3^+^+MUM1^+^) [IE+ST] were analyzed using original data value. The remaining variables were analyzed using the data from ln transformation. Median numbers (interquartile range) of CD20^+^ B-cells per 1mm^2^ of tumor area (intraepithelial [IE] plus stroma [ST]) and IE compartment within tumor area were 590 (224-1276) and 101 (38-109), respectively; the corresponding numbers of MUM1^+^ plasma cells were 298 (180-605), and 67 (22-145), respectively. The proportion of MUM1^+^ plasma cells among all TILs (MUM1^+^ cells/[CD3^+^ cells+CD20^+^ cells+MUM1^+^ cells] x 100) ranged from 1% to 59% (median 13%) in the tumor area and showed a significant association with OS by univariate Cox analysis (negative correlation with hazard ratio (HR)=12.50 [95% confidence interval (CI), 1.75-89.27]).

### Univariate cox proportional hazards analysis

In addition to pathologic TNM tumor stage, treatment modalities, and age at diagnosis, the following TIL variables had statistically significant associations with OS: CD20^+^ [IE], CD20^+^/CD3^+^ [IE] and MUM1^+^/(CD20^+^+CD3^+^+MUM1^+^) [IE +ST] as continuous variables. The lower count of CD20^+^ [IE], the lower ratio of CD20^+^/CD3^+^ and the higher ratio of MUM1^+/^(CD20^+^+CD3^+^+MUM1^+^) [IE+ST] were significantly associated with shorter OS (Table 2).

Plots of the martingale residuals and Contal and O'Quigley indicated cut-off points to dichotomize CD20^+^ [IE] (adjusted p=0.067), CD20^+^/CD3^+^ [IE] (adjusted p=0.139) and MUM1^+^/(CD20^+^+CD3^+^+MUM1^+^) [IE+ST] (adjusted p=0.023). Therefore, these three variables were modeled as both continuous and dichotomizing variables in further survival analyses (Table 3). For the remaining variables, no cut-off point related to overall survival could be defined, and were assessed as original continuous variables only (Table 2).

For CD20^+^[IE], there were 5 significant cut-off points between 84.65 and 91.77, with 85.66 being the most informative value via the log rank statistic test. For MUM1^+^/(CD20^+^+CD3^+^+MUM1^+^) [IE+ST], 10 statistically significant cut-off points were found between 21.32% and 25.55%, with the most informative value at 24.79%.

### Multivariate cox proportional hazards analyses

Three TIL variables proved to be significant prognostic factors in univariate Cox analysis using dichotomous values, CD20^+^ [IE], CD20^+^/CD3^+^ [IE] and MUM1^+^/(CD20^+^+CD3^+^+MUM1^+^) [IE+ST], were converted into different dichotomous variables in a range of potential cut-off points and were further assessed. The estimated HR and associated 95% CI were presented in supplemental Table 1, with age at diagnosis and treatment adjusted in all models.

**Figure 2:**
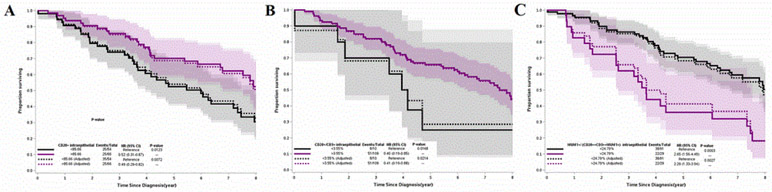
Survival curves for resected lung adenocarcinomas according to risk groups based on the best cut-off points of CD20^+^[IE] (A), CD20^+^ CD3+[IE] (B), and MUM1^+^/ (CD20^+^+CD3^+^+MUM1^+^) in tumor area (IE plus ST) (C). The multivariate Cox Proportional Hazards Curves were adjusted with treatment modalities and age at diagnosis. IE, intraepithelial compartment of tumor; ST, stromal compartment of tumor

The higher count of CD20^+^ [IE] remained an independent favorable prognostic factor at a cutoff ranging from 75.49 to 101.55. The largest difference of survival was seen when the count was greater than 85.66 (HR=0.49; 95% CI=0.29-0.83, p=0.007) (Figure 1). CD20^+^[IE] as a continuous variable also showed a significant association with favorable prognosis (HR=0.83; 95% CI=0.69-0.99, p=0.048) in multivariate analysis. The higher ratio of CD20+/CD3^+^ [IE] also remained an independent prognostic factor at a cutoff ranging from 3.55% to 11.6% (Figure 2).

The higher ratio of MUM1^+^/(CD20^+^+CD3^+^+MUM1^+^)[IE+ST] was an independent factor of worse prognosis at a cutoff ranging from 21.1% and 26.0% in multivariate analysis and the largest difference of survival occurred at a cutoff of 24.79% (HR=2.29; 95% CI=1.33-3.94, p=0.003) (Figure 1). It was also an independent negative prognostic factor when treated as a continuous variable (HR= 10.45, 95% CI, 1.33-81.88, p=0.033).

## DISCUSSION

In the present study, we found that the increased plasma cell % among TILs in the tumor area (IE+ST) correlated with worse OS, especially if it is greater than 25%. On the other hand, the increased count of IE B-cell and the ratio of IE B- and T-cells were associated with better OS. The localization of B-cells and plasma cells within the tumor affected their prognostic significance in our study. A few previous studies reported the various effects of B-cells and plasma cells in NSCLC or adenocarcinomas of the lung.

Kurebayashi et al. performed a comprehensive immunoprofiling on 111 lung adenocarcinoma cases. They reported that there were infiltrating immune cells composed of four distinct immunosubtypes: CD8, mast cells, macrophage/dendritic cells, and plasma cells and found that plasma cell as an independent negative prognostic factor [6]. They postulated that it is mediated by immunosuppressive cytokine IL-35 produced by these plasma cells.

Lohr et al. analysed the B-cell and plasma cell markers along with immunoglobulin kappa C (IGKC) expression in NSCLC using immunohistochemistry on a tissue microarray [5]. They reported that IGKC protein expression was independently associated with longer survival, with particular impact in the adenocarcinoma cases in their cohort of NSCLC patients. A comparable association with survival was seen with CD138+ plasma cells but not with CD20^+^ B-cells. Based on these results, they concluded that IGKC expression in stroma-infiltrating plasma cells is a positive prognostic marker in NSCLC. On the other hand, Al-Shibli et al. reported that plasma cells were not prognostic indicators in their cohort of 335 NSCLC cases based on tissue microarray [7].

There have also been some studies on the role of B cells and plasma cells in tumor immunity in solid tumors other than lung cancers. A few studies showed tumor-infiltrating B cells are associated with favorable prognosis in ovarian cancer, which is in line with our results [13,14]. They postulated that this phenomenon may be due to the fact that depletion of B cells impairs the T-cell-dependent antitumor cytotoxic response [14]. In regard to the role of plasma cells, a few studies reported that plasma cells had an immunosuppressive role in prostatic cancers, also in keeping with our results; IgA+ plasma cells within tumors induced CD8^+^ cell exhaustion and suppressed anti-tumor cytotoxic T cell responses through PD-L1 and IL-10, either of which could result in anergy or exhaustion [15-17].

The reason for the conflicting results in the literature is not entirely clear. It might be partly due to the methodology such as full section vs. tissue microarray, different tumor types (NSCLC cases including squamous cell carcinomas, large cell carcinomas as well as adenocarcinomas, vs. pure adenocarcinoma cases), analysis considering different tumor compartments (e.g. IE, ST, IE+ ST, etc.), quantitation methods (DIA vs. manual), for example.

In our study, we performed DIA using whole sections from a one year (2009) cohort to ensure the homogeneous treatment and tissue quality as well as the sufficient follow up information. We carefully chose the five representative boxes for annotation encompassing both central and peripheral portions of tumor. We also did methodical QA to ensure the accuracy of quantitation. We analyzed the TILs within the entire box (IE +ST) and the TILs within the epithelial element of tumor (IE), which may exert different role(s) in tumor microenvironment. Previous studies on plasma cells within TILs used CD138 that has been well known to cross react with epithelial cells including the lung carcinoma cells and might have caused some difficulty in counting either by manual or DIA methods. Thus, we used MUM1 antibody to avoid such problem, which allowed us a more accurate counting of plasma cells.

We also analyzed the TILs in different compartments by using original value or ln converted value for those with outliers, to ensure appropriate statistical analysis. We also tried to find if there is any dichotomous number showing the significance in the survival analysis. There were multiple valid dichotomous cut offs, which are shown in the supplemental Table 1.

## CONCLUSION

The main limitations of this study are the retrospective nature and relatively small number of cases included in the analysis. We also could not provide with mechanistic information for the results. Further studies with more comprehensive markers and molecular approaches would be warranted to better understand the role of humoral immunity in tumor microenvironment.

## Supplementary Material

Suppl Table

## Figures and Tables

**Table 1:** Descriptive characteristics of 120 resected lung adenocarcinomas.

Clinical characteristics		Tumor-infiltrating immune cell characteristics		Ratio of tumor-infiltrating immune cell characteristics	
Age, yrs		CD 20 [IE+ST] ln#		MUM1/(CD2+CD3+MUM1) [IE+ST]	
Median	68.5	Mean (SD)	5.8 (1.1)	Mean (SD)	0.2 (0.1)
Range	46.0-91.0	Median	5.7	Median	0.1
Sex		Range	(2.1-8.2)	Range	(0.0-0.6)
Male	52 (43.3%)	CD 20 [IE] ln#		MUM1/(CD2+CD3+MUM1) [IE] ln#	
Female	68 (56.7%)	Mean (SD)	4.0 (1.3)	Mean (SD)	−2.5 (1.1)
Current	31 (25.8%)	Median	4.2	Median	−2.4
Tumor stage		Range	(0.1-7.0)	Range	(−5.8-−0.5)
Stage I	82 (68.3%)	MUM1 [IE+ST] ln#		CD20/CD3 [IE+ST] ln#	
Stage II	17 (14.2%)	Mean (SD)	5.8 (1.1)	Mean (SD)	−1.0 (1.0)
Stage III/IV	21 (17.5%)	Median	5.7	Median	−1
Treatment		Range	(2.1-8.2)	Range	(−3.9-1.4)
Only surgery	90 (75.0%)	MUM1 [IE] ln#		CD20/CD3 [IE] ln (n =119)*#	
Surgery^+^chemoradiation	30 (25.0%)	Mean (SD)	4.0 (1.3)	Mean (SD)	−1.7 (1.1)
Cigarette smoking status		Median	4.2	Median	−1.7
Never	25 (20.8%)	Range	(0.1-7.0)	Range	(−4.9-0.8)
Former	64 (53.3%)				
Current	31 (25.8%)				
Vital status					
Alive	60 (50.0%)				
Dead	60 (50.0%)				

**Abbreviations**: SD: Standard deviation; Q1: Upper quartile; Q3: Lower quartile; ln: Natural logarithm; [IE]: Intraepithelial compartment of the tumor; [IE+ST]: Both intraepithelial and stromal compartments of the tumor.

* : The variable with an outlier.

# : The variable analyzed using ln transformation data.

**Table 2:** Univariate and multivariate cox proportional hazard models for the predictors of overall survival (continuous variables).

Variables	Univariate analysis			Multivariate analysis*		
	HR	95% CI	p value	HR	95% CI	p value
CD 20 [IE]	0.81	0.68-0.96	0.0158	0.83	0.69-0.99	0.048
CD20 [IE+ST]	0.94	0.77-1.15	0.5588	-	-	-
CD20/CD3 [IE]	0.74	0.59-0.94	0.0137	0.77	0.61-0.99	0.045
CD20/CD3 [IE+ST]	0.89	0.67-1.18	0.4068	-	-	-
MUM1 [IE]	0.94	0.78-1.15	0.5733	-	-	-
MUM1 [IE+ST]	1.14	0.89-1.47	0.3024	-	-	-
MUM1/CD3+CD20+MUM1 [IE]	0.94	0.72-1.23	0.6696	-	-	-
MUM1/CD3+CD20+MUM1 [IE+ST]	12.5	1.75-89.27	0.011	10.45	1.33-81.88	0.033

**Abbreviations:** IE: Intraepithelial compartment of the tumor; IE+ST: Both intraepithelial and stromal compartments of the tumor; HR: Hazard Ratio; CI: Confidence Interval.

* : Treatment modalities and age were adopted as covariates in each multivariate analysis.

**Table 3:** Univariate and multivariate cox proportional hazard models for the predictors of overall survival (dichotomous variables based on the best cutoffs*).

		Univariate analysis		Multivariate Analysis†		
Variables	HR	95% CI	p value	HR	95% CI	p value
CD 20 [IE] >85.66/1 mm^2^ vs. <85.66/1mm_2_	0.52	0.31-0.87	0.0123	0.49	0.29-0.83	0.007
CD20/CD3 [IE] >3.55% vs. <3.55%	0.40	0.19-0.85	0.0168	0.41	(0.19-0.88)	0.0214
MUM1/CD3+CD20+MUM1 [IE+ST] >24.79% vs. <24.79%	2.65	1.56-4.49	0.0002	2.29	1.33-3.94	0.003

**Abbreviations:** IE: Intraepithelial compartment of the tumor; IE+ST: Both intraepithelial and stromal compartments of the tumor; HR: Hazard Ratio; CI: Confidence Interval.

* : The best cutoffs were based on the lowest p-value by log rank statistic test for overall survival, among the potential cutoffs determined by plots of the martingale residuals and Contal and O'Quigley.

† : Treatment modalities and age were adopted as covariates in each multivariate analysis.

## ABBREVATIONS:

TIL: Tumor-Infiltrating Lymphocyte
NSCLC: Non-Small Cell Lung Cancer
OS: Overall Survival
IHC: Immunohistochemistry
IE: Intraepithelial Compartment
ST: Stromal Compartment
HR: Hazard Ratio
CI: Confidence Interval
