# Supplementary material for: Increased Plasma Cells and Decreased B-cells in Tumor Infiltrating Lymphocytes are Associated with Worse Survival in Lung Adenocarcinomas
Source: J Clin Cell Immunol. Author manuscript; Available in PMC 2020 Jun 10. (PMC7286600)
Supplement: Suppl Table [file NIHMS1566841-supplement-Suppl_Table.pdf]

Supplemental Table 1. Multivariate Cox proportional hazards analysis for overall survival based on the potential cutoffs*

CD20$^+$[IE] †			CD20$^+$/CD3$^+$[IE]			MUM1$^+$/ (CD20$^+$+CD3$^+$+MUM1$^+$) [IE+ST]		
Parameter	HR (95% CI)	*p*	Parameter	HR (95% CI)	*p*	Parameter	HR (95% CI)	*p*
>72.13	0.63(0.37-0.04)	0.070	>3.00%	0.48(0.21,1.07)	0.073	>20.4%	1.65(0.98-2.79)	0.063
<72.13	--		<3.00%%	-		<20.4%		
>75.49	0.59(0.36-0.99)	0.045	>3.55%	0.41(0.19,0.88)	0.021	>21.1%	1.71(1.01-2.90)	0.047
<75.49	--		<3.55%	-		<21.1%	--	
>85.66	0.49 (0.29,0.83)	0.007	>7.13 %	0.46 (0.26,0.81)	0.012	>24.79%	2.29 (1.33,3.94)	0.003
<85.66	--		<7.13%	--		<24.79%	--	
>101.55	0.57(0.34,0.96)	0.034	>11.60%	0.57(0.34,0.97)	0.038	>26.0%	2.00(1.07-3.75)	0.031
<101.55	--		<11.60%	--		<26.0%	--	
>101.80	0.65(0.39,1.09)	0.105	<11.72%	0.63(0.37,1.07)	0.0852	>26.4%	1.83(0.97-3.45)	0.064
<101.80	--		<11.72%	--		<26.4%	--	

Abbreviations: [IE], intraepithelial compartment of the tumor; [IE+ST], both intraepithelial and stromal compartments of the tumor; HR, hazard ratio; CI, confidence interval.

*, the potential cutoffs were determined by plots of the martingale residuals and Contal and O'Quigley.
